# Association of Metabolically Healthy and Unhealthy Obesity Phenotype with Markers Related to Obesity, Diabetes among Young, Healthy Adult Men. Analysis of MAGNETIC Study

**DOI:** 10.3390/life11121350

**Published:** 2021-12-07

**Authors:** Mateusz Lejawa, Kamila Osadnik, Zenon Czuba, Tadeusz Osadnik, Natalia Pawlas

**Affiliations:** 1 Department of Pharmacology, Faculty of Medical Sciences in Zabrze, Medical University of Silesia, Jordana 38 Str., 41-808 Zabrze, Poland; kosadnik@sum.edu.pl (K.O.); tadeusz.osadnik@sum.edu.pl (T.O.); natalia.pawlas@sum.edu.pl (N.P.); 2 Department of Microbiology and Immunology, Faculty of Medical Sciences in Zabrze, Medical University of Silesia, Jordana 19 Str., 41-808 Zabrze, Poland; zczuba@sum.edu.pl

**Keywords:** adipokines, metabolically healthy obesity, metabolically unhealthy obesity, biomarkers

## Abstract

Adipose tissue secretes many regulatory factors called adipokines. Adipokines affect the metabolism of lipids and carbohydrates. They also influence the regulation of the immune system and inflammation. The current study aimed to evaluate the association between markers related to obesity, diabesity and adipokines and metabolically healthy and unhealthy obesity in young men. The study included 98 healthy participants. We divided participants into three subgroups based on body mass index and metabolic health definition: 49 metabolically healthy normal-weight patients, 27 metabolically healthy obese patients and 22 metabolically unhealthy obese patients. The 14 metabolic markers selected were measured in serum or plasma. The analysis showed associations between markers related to obesity, diabesity and adipokines in metabolically healthy and unhealthy obese participants. The decreased level of adipsin (*p* < 0.05) was only associated with metabolically healthy obesity, not with metabolically unhealthy obesity. The decreased level of ghrelin (*p* < 0.001) and increased level of plasminogen activator inhibitor-1 (*p* < 0.01) were only associated with metabolically unhealthy obesity, not with metabolically healthy obesity. The decreased level of adiponectin and increased levels of leptin, c-peptide, insulin and angiopoietin-like 3 protein were associated with metabolically healthy and unhealthy obesity. In conclusion, our data show that metabolically healthy obesity was more similar to metabolically unhealthy obesity in terms of the analyzed markers related to obesity and diabesity.

## 1. Introduction

Obesity is a global health problem that has grown into an epidemic [1]. From 1975 to 2016, the number of obese people in the world tripled. In 2016, more than 1.9 billion adults (18 and over) were overweight. Of these, 650 million were obese. Overall, about 13% of the world’s adult population (11% men and 15% women) were obese in 2016 [2].

Metabolic syndrome (MetS), a recognized complication of obesity, is associated with an increased risk of diabetes and hypertension. Numerous studies indicate that insulin resistance, chronic inflammation, adipokines and increased oxidative stress play essential roles in its pathogenesis [3,4,5]. In some patients, despite the presence of risk factors, MetS does not develop. This condition is called metabolically healthy obesity (MHO). There are no universally accepted criteria for defining MHO. In most MHO definitions, the patient is obese and does not have metabolic abnormalities such as dyslipidaemia, impaired glucose tolerance, or risk factors for MetS [6,7,8]. People with MHO show a reduced amount of visceral fat, smaller adipocytes and a lower level of inflammation than obese people who are metabolically unhealthy (MUO) [9]. The term MHO was first used in the 1980s [10]. Epidemiological studies suggest that this condition may range from 18% to 44% depending on the given population (18% among Korean women, 44% among Americans) [10]. Such significant differences result from different criteria adopted for the determination of the MHO. It seems that MHO more often affects women than men. Moreover, MHO status declines with age in both sexes [11,12]. Most researchers agree that obesity should be defined in subjects with a BMI ≥ 30 [12]. Until now, different definitions of the MHO phenotype have been encountered in many studies. These definitions are based on biochemical parameters, anthropometric measurements, or the insulin resistance. The use of non-identical definitions of MHO is the main limiting factor, making it difficult to appropriately compare data between numerous observational studies and draw correct interpretations regarding the association of MHO, CVD and mortality [13,14]. The causes of this condition are not fully known yet. Some authors believe that MHO precedes the development of MUO. Therefore, it is advisable to research markers of inflammation, insulin resistance or those regulating the state of hunger and satiety in MHO, MUO and metabolically healthy slim people to explain this condition [7]. Assuming that changes in the concentrations of the analyzed markers precede the appearance of MUO and then MetS, assessing their concentrations in these groups of patients will allow us to verify the hypothesis that MHO is a state preceding the development of MUO.

The present study aimed to determine the association among markers related to obesity, diabesity, adipokines and metabolically healthy and unhealthy phenotypes in healthy young men.

## 2. Materials and Methods

### 2.1. Study Participants

Subjects were recruited from the Metabolic and Genetic Profiling of Young Adults with and without a Family History of Premature Coronary Heart Disease (MAGNETIC) study. The purpose of the MAGNETIC study was to define classical and genetic risk factors for coronary artery disease (CAD) in healthy young adults with and without a familial history of premature coronary artery disease (P-CAD). For this purpose, descendants of patients hospitalized at the Silesian Center for Heart Disease due to coronary artery disease and a control group of healthy volunteers were invited to participate in the project. The detailed study protocol has already been described by Osadnik et al. [15].

The study population included 98 healthy Caucasian men (49 slim and 49 obese). We conducted our research exclusively on males to ensure the homogeneity of the study group. Sex differences affect the level of the analyzed parameters. The selected population was divided into three groups based on their BMI value and metabolic health status. No participants in the study were on lipid-lowering medication, hypertensive therapy or drug treatment for type 2 diabetes (T2D). Obesity is defined as BMI ≥ 30 kg/m^2^ according to the current definition World Health Organization (WHO). The definition of metabolic health is taken from the guidelines of the International Diabetes Federation (IDF) [16] and the third report of the National Cholesterol Education Program Adult Treatment Panel III (NCEP-ATP III) [17]. Patients with ≤2 components of metabolic syndrome were considered metabolically healthy: (1) fasting blood glucose ≥100 mg/dL or current use of blood glucose-lowering agents; (2) blood pressure ≥130/85 mmHg or current use of blood pressure-lowering agents; (3) triglyceride levels ≥150 mg/dL or current use of lipid-lowering agents; (4) high-density lipoprotein cholesterol <40 mg/dL in men; and (5) waist circumference in men >102 cm. Metabolically healthy normal weight participants (MHNW) were defined as a participants with average weight (18.5 to 24.99 kg/m^2^) and metabolic health.

### 2.2. Measurement of Anthropometric and Biochemical Parameters

During first visits at the Silesian Center for Heart Disease, participants’ height, weight, waist circumference, hip circumference, systolic blood pressure (SBP) and diastolic blood pressure measurements (DBP) were measured by trained examiners.

Venous blood samples were collected after an overnight (8–10 h) fast using S-Monovette tubes coated with EDTA (whole blood or plasma), S-Monovette tubes with clotting activator (serum) or S-Monovette tubes with 3.1% sodium citrate (for fibrinogen content). The blood samples were centrifuged at 1500 rpm for 10 min for serum or 3000 rpm for 10 min for plasma in a refrigerated centrifuge from Eppendorf (Hamburg, Germany) at 4 °C. Total cholesterol (TC), low-density lipoprotein cholesterol (LDL-C), high-density lipoprotein cholesterol (HDL-C), triglycerides (TG), apolipoprotein A1 (apoA1), apolipoprotein B (apoB), lipoprotein(a) (Lp(a)), high-sensitivity C-reactive protein (hsCRP), glucose, bilirubin, uric acid, ceruloplasmin, fibrinogen concentrations in serum and glycated haemoglobin (HbA1c) levels in whole blood were measured by standard laboratory techniques in Central Laboratory Silesian Centre for Heart Diseases in Zabrze as previously described [15,18]. Serum and plasma, which were not used for the determination of biochemical parameters, were aliquoted into polypropylene 1.5 mL tubes and immediately placed in a −80 °C freezer. Samples were stored in a monitored freezer until testing. Samples were thawed only before testing.

Visceral adiposity index (VAI) was calculated based on the formula previously proposed by Amato et al. [19].

Homeostasis model assessment (HOMA-IR) was calculated based on the formula previously proposed by Matthews et al. [20].

### 2.3. Measurement of Markers Related to Obesity and Diabesity

Adiponectin, adipsin, leptin, visfatin, resistin, plasminogen activator inhibitor-1 (PAI-1), ghrelin, gastric inhibitory peptide (GIP), c-peptide, glucagon-like peptide-1 (GLP-1), glucagon and insulin were measured in serum per manufacturer’s specifications using magnetic bead-based multiplex assays from Bio-Rad (Minneapolis, MN, USA). Plasma level of angiopoietin-like 3 protein (ANGPTL3) and angiopoietin-like 4 protein (ANGPTL4) were measured using magnetic bead-based multiplex reagents from the R&D System (Minneapolis, MN, USA). Levels of analytes were measured using a Bio-Plex 200 System from Bio-Rad. All samples were tested in duplicate. Concentrations of analytes were calculated using Bio-Plex Manager software (version 5.0).

### 2.4. Statistical Analysis

Statistical analysis was carried out using the R language in Rstudio (R software version 3.6.1) [21]. Continuous variables are expressed as the median and 25th–75th percentiles. Categorical variables (also called qualitative variables) are expressed as absolute (n) and relative (%) frequencies. To check if the data were approximately normally distributed, we used graphical methods to analyze the normal probability plots (quantile–quantile plots). After review, the majority of data were found to be non-normal distributed. Due to this fact and the small size of the study group, we used non-parametric tests in further analyses. To detect outliers in data, we investigated the box plot of continuous variables. We considered values above or below the whiskers in the box plot as outliers. We treated outliers as missing values. According to metabolic health status, differences between the three groups were compared by the Kruskal–Wallis test and Jonckheere’s trend test (continuous variable). The comparison of categorical variables among the groups was performed using the chi-squared test and Cochran–Armitage test for trend. We also performed Spearman’s correlation analysis between markers related to obesity and diabesity to identify which are dependent on each other. In the next step to determine relations between markers related to obesity and diabesity with metabolic health status, we used univariate and multivariate regression analyses based on a general linear model. There were some missing data for ANGPTL3 (9.18%), ANGPTL4 (9.18%), ceruloplasmin (3.06%), ghrelin (2.04%), GIP (2.04%), GLP-1 (6.12%), glucagon (6.12%), PAI-1 (2.04%), SBP (1.02%), DBP (2.04%), waist–-hip ratio (WHR) (1.02%), VAI (3.06%) and visfatin (1.02%). Before performing regression analysis, missing values were imputed using the machine learning-based data imputation algorithm missForest [22]. Moreover, for selected non-normal distributed variables, we performed log transformations to obtain a normal distribution. A *p* value below 0.05 was considered as statistically significant.

## 3. Results

### 3.1. Demographic, Biochemical and Anthropometric Parameters

The baseline characteristics of the subject classified by metabolic status are shown in Table 1, Table 2 and Table 3. We did not notice any differences between groups related to age, family history of DM, smoking, sleeping habits or physical activity. We observed significant differences between the groups concerning family history of P-CAD, BMI, WHR, VAI, SBP, DBP, hsCRP, fibrinogen, TC, HDL-C, HDL%, LDL-C, TG, apoA1, apoB, HbA1c, glucose, HOMA-IR, bilirubin, uric acid, ceruloplasmin, adiponectin, adipsin, leptin, leptin/adiponectin ratio, ghrelin, c-peptide, insulin and AGPTL3. MHO participants had higher BMI values, WHR, VAI, SBP, DBP, hsCRP, TC, LDL-C, TG, apoB, HbA1, glucose, HOMA-IR, uric acid, ceruloplasmin, leptin, leptin/adiponectin ratio, c-peptide, insulin and ANGPTL3 and lower HDL-C, HDL%, apoA1, bilirubin, adiponectin, adipsin and ghrelin compared to the MHNW group (*p* < 0.05). MUO participants had higher BMI values, waist circumference, WHR, VAI, SBP, DBP, hsCRP, fibrinogen, TC, LDL-C, TG, apoB, HbA1, glucose, HOMA-IR, uric acid, ceruloplasmin, leptin, leptin/adiponectin ratio, c-peptide and insulin and lower HDL-C, HDL%, apoA1, bilirubin, adiponectin, ghrelin compared to the MHNW group (all *p* < 0.05). Compared to MUO, the MHO group had higher HDL-C, HDL% but lower BMI, WHR, DBP, hsCRP, fibrinogen, TG, apoB, c-peptide and ANGPTL3 (*p* < 0.05).

**Figure 1:**
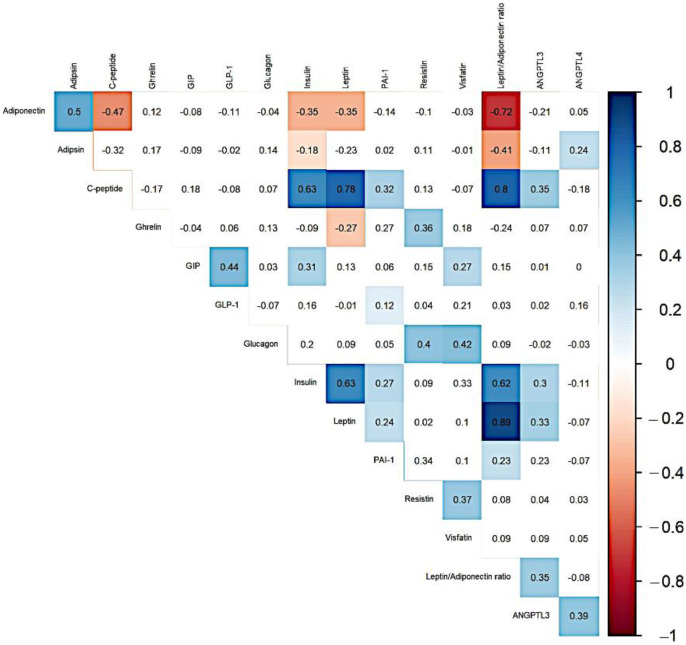
Correlogram of markers related to obesity and diabesity. Only significant Spearman’s correlation coefficients shown.

The correlation analysis (Figure 1) showed a significant positive correlation between the markers of obesity and diabesity—adiponectin: adipsin; adipsin: ANGPTL4; c-peptide: insulin, leptin, PAI-1, leptin/adiponectin ratio, ANGPTL3; ghrelin: resistin; GIP: GLP-1, insulin, visfatin; GLP-1: PAI-1; glucagon: resistin, visfatin; insulin: leptin, PAI-1, leptin/adiponectin ratio, ANGPTL3; leptin: PAI-1, leptin/adiponectin ratio, ANGPTL3; PAI-1: leptin/adiponectin ratio; resistin: visfatin; leptin/adiponectin ratio: ANGPTL3; and ANGPTL3: ANGPTL4. There were significant negative correlations found between adiponectin: c-peptide, insulin, leptin; adipsin: insulin, leptin/adiponectin ratio; and ghrelin: leptin.

**Figure 2:**
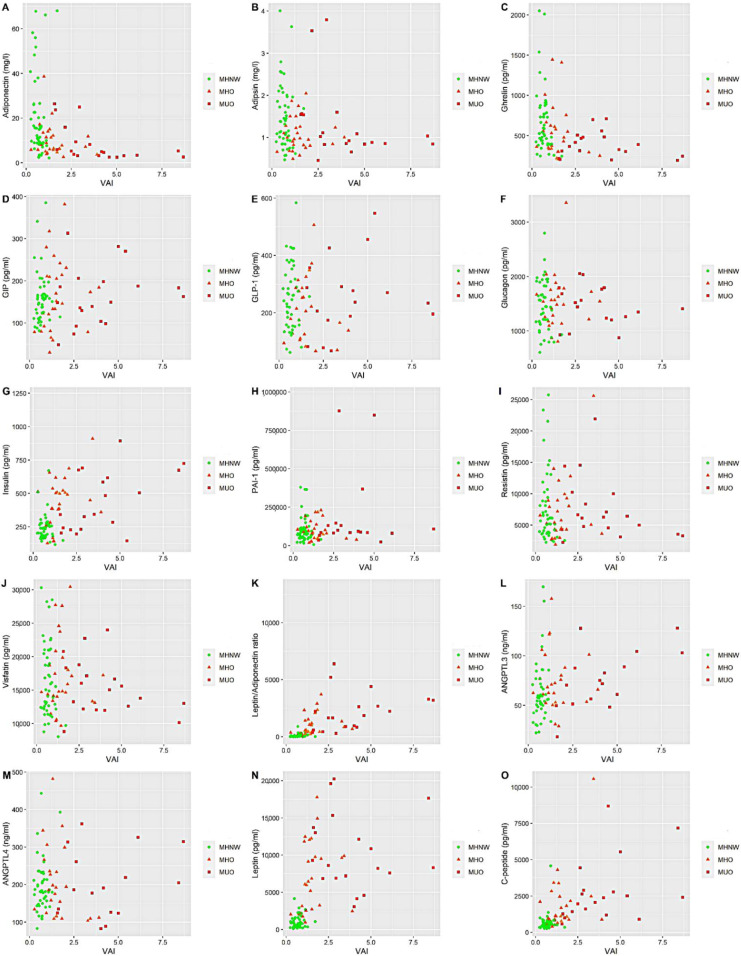
The connection between visceral adiposity index and markers related to obesity and diabesity—differences between men with different metabolic status. MHNW: metabolically healthy normal weight; MHO: metabolically healthy obese; MUO: metabolically unhealthy obese; VAI: visceral adiposity index; GIP: gastric inhibitory peptide; GLP-1: glucagon-like peptide-1; PAI-1: plasminogen activator inhibitor-1; ANGPTL3: angiopoietin-like 3 protein; ANGPTL4: angiopoietin-like 4 protein. Data are presented in plots where the y-axis shows the concentration of the selected marker, and the x-axis shows the visceral adiposity index value. (**A**) adiponectin; (**B**) adipsin; (**C**) ghrelin; (**D**) GIP; (**E**) GLP-1; (**F**) glucagon; (**G**) insulin; (**H**) PAI-1; (**I**) resistin; (**J**) visfatin; (**K**) leptin/adiponectin index; (**L**) ANGPTL3; (**M**) ANGPTL4; (**N**) leptin; and (**O**) c-peptide.

In the following section of the work, we present how the changes in the concentration of selected markers change the VAI value (a reliable indicator of visceral fat function associated with cardiometabolic risk) (Figure 2). We wanted to observe whether the analyzed patients grouped in the graph were well-limited, independent groups in terms of their metabolic status. After analyzing the plots, it can be seen that the MHO group is not well limited compared to MHNW and MUO. It seems to be an intermediate state towards MUO.

### 3.2. Relationship between Markers Related to Obesity, Diabesity and Metabolic Health Status

Univariate regression analyses indicate that MHO and MUO, compared to MHN, were associated with a decreased adiponectin level, increased level of leptin, c-peptide, insulin, ANGPTL3, and a higher value of leptin/adiponectin ratio. MUO was associated with a decreased level of ghrelin (*p* < 0.001) and an increased level of PAI-1 (*p* < 0.05). Additionally, this analysis showed that MHO was associated with a decreased level of adipsin (*p* < 0.05) (Table 4).

These markers, which were significantly related in univariate analysis, remained significantly associated with MHO or MUO after adjustment for age, smoking and alcohol consumption (Table 5).

## 4. Discussion

Obesity is undoubtedly related to diabetes, dyslipidemia, hypertension, chronic inflammation and metabolic syndrome [23]. All of these obesity comorbidities increase the risk of CVD. However, not all obese people experience metabolic disorders. We call such people metabolically healthy obese. Unlike MHO, there are also obese individuals with unhealthy metabolic obesity and an increased risk of collapse for cardiometabolic diseases [24]. MHO is an intermediate risk state. The excessive risk of obesity-related diseases is not as high as in MUO but still higher than in MHNW. However, when examining how these people change over time, research shows that MHO is confusing. MHO often turns into MUO due to the presence of metabolic risk factors and often leads to other types of diseases [25]. MHO, therefore, is best viewed only as a state of relative health, not absolute health. Identifying such patients is essential because appropriate nutritional interventions could stop the process, reducing cardiovascular risk in these patients, among other risks [26]. In our work, we wanted to investigate the relationship between obesity and diabesity markers (with an emphasis on adipokines) not widely used in clinical practice in the group of people with different metabolic health statuses and weights.

As in the work of Aguilar-Salinas et al. and Liu et al., we showed higher levels of adiponectin in MHO vs. MUO [27,28]. Both studies measured the level of total adiponectin but not the high molecular weight (HMW) adiponectin. HMW adiponectin is more strongly associated with susceptibility markers for insulin or MetS than total adiponectin [29,30]. Additionally, in our work, we recorded total adiponectin. The studies mentioned above confirmed the relationship between adiponectin and a metabolically healthy obesity phenotype. This is because adiponectin plays a significant protective role in the pathogenesis of obesity-related disorders [31]. Additionally, hypoadiponectinemia is a predictor of the development of arterial hypertension and a risk factor for atherosclerosis [32,33].

However, in both studies, MHNW and MHO have similar adiponectin levels, contrary to our results. Moreover, MHNW had a higher level than MUO, but the differences in concentration were not as significant as in our study. The participants in these studies were older than those in our work (about 43 years old). Perhaps with age, the considerable differences in adiponectin levels between groups decrease. However, higher adiponectin levels (MHNW and MHO vs. MUO) remain associated with metabolic health. Our results also confirm that in people with MHO, leptin and adiponectin levels are intermediate between MHNW and MUO [34,35]. Leptin, which is secreted by adipose tissue, plays an essential role in controlling fat stores in the body through the coordinated regulation of appetite, metabolism and the body’s energy balance [36]. Increased circulating leptin levels are typical in obesity and are independently associated with CVD risk in humans [37]. In addition, we also confirmed that the leptin/adiponectin ratio in MHO adults was intermediate between MHNW and MUO. Similar results were shown by Ding et al. in their work, where the same leptin/adiponectin ratio was maintained in children and adolescents [38]. Our study did not confirm a significantly higher level of leptin in MUO vs. MHO. The work of Jamar et al. partially demonstrates our results. However, in contrast to their results, in our work MHO and MUO differed in their leptin/adiponectin ratios, not just their levels of leptin [39].

MHO and MUO subtly differ in chronic inflammation and insulin resistance. Our study found that MHO has similar insulin resistance but differs in the chronic inflammation level compared to MUO. Our results stand in contrast to those of Molli et al., which show the opposite outcome [40]. Interestingly, Marques-Vidal et al. found that MHO individuals had lower hsCRP levels than MUO, depending on the definition used for MHO [41]. Our MHO definition coincides with theirs.

The use of univariate regression followed by multivariate regression showed that among the tested markers, only adipsin is related to MHO compared to MUO. Adipsin, also called complement factor D, activates the alternative complement pathway involved in the humoral response to infectious agents. Moreover, it exhibits properties that induce the differentiation of adipocytes and the synthesis of triglycerides [42]. It also increases insulin secretion in response to increased glucose levels after eating a meal [43]. In our work, the MHO patients had a lower level of this adipokine compared to those with MUO and MHNW. The role of adipsin in people with metabolic health obesity remains largely elusive and has not been studied in the context of metabolic obesity. According to other studies, low adipsin levels are associated with a higher risk of T2D [43,44,45]. On the other hand, a higher level of adipsin in MUO can be treated as a compensatory mechanism to improve the metabolism of glucose and lipids [46]. Lo et al. demonstrated that mice with an adipsin deficiency exhibited reduced inflammation in adipose tissue. These findings suggest that higher adipsin levels might contribute to inflammation, which increases in metabolic disorders [44]. To our knowledge, there are no other studies on the exact molecular mechanisms of circulating adipsin in MHO, so future studies in vivo and in vitro should be done to address this issue.

In our work, we did not show any relationship between a higher concentration of resistin and the presence of MHO or MUO. According to our results, Ambrosi et al. also found that resistin, leptin and adiponectin levels were almost similar in both MHO and MUO [47]. Christou et al. found different results to our work. Circulating resistin was higher in MUO compared with MHO [48]. The main sources of resistin in humans are adipocytes and immune system cells. Experiments on animals have shown that resistin is involved in inducing insulin resistance. A study on obese mice showed that the serum level of resistin was significantly higher than that of its lean counterparts. In humans, the relationship between resistin level and the development of insulin resistance is ambiguous. Some studies show a significant relationship between resistin concentration and insulin resistance, while others do not confirm such a relationship. The analysis of the results of numerous experiments has shown that resistin can play a role in inflammatory processes. It was also found that resistin increases the expression of IL-6 and TNF-alpha [49,50].

In turn, MUO patients are characterized by a lower concentration of ghrelin and a higher concentration of PAI-1 than MHNW and MHO. A recent study evaluated the levels of ghrelin in MHO, MUO and non-obese groups. In that study, the MHO and MUO groups had a lower level of ghrelin when compared to MHNW. Additionally, the level of ghrelin was similar between MHO and MUO [51]. Ferrer et al. found dissimilar results to our work when comparing “morbidly” MHO and MUO (BMI > 40 kg/m^2^) in a Spanish population. They found a higher level of ghrelin in MUO than in MHO [52]. Ghrelin is a peptide hormone secreted by enteroendocrine cells of the gastrointestinal tract, especially of the stomach. It facilitates the consumption of food by increasing the secretion of gastric acid or improving its motility. Obese people have a lower concentration of ghrelin compared to lean people [53,54].

PAI-1, in turn, is a protein that is an inhibitor of PA, the enzyme responsible for the conversion of plasminogen to plasmin. Elevated PAI-1 concentration promotes a state of hypofibrinolysis, which conditions a greater risk of thrombosis and atherosclerosis [55,56]. Our results confirm the relationship already described in the literature between a higher concentration of PAI-1 in MUO compared to MHO [34,57].

ANGPTL3 and ANGPTL4 are proteins whose malfunction is related to dyslipidaemia or insulin resistance [58,59]. Compared to MHNW, only increased levels of ANGPTL3 were associated with both MHO and MUO in our work. In turn, the level of ANGPTL4 was elevated in all obese subgroups but at similar level. Our results are partially in line with those of Schinzari et al. In the work Schinzari et al., the level of circulating ANGPTL3 was elevated in MUO subjects compared to lean subjects. However, contrary to our results, this was not the case for MHO. ANGPTL4 was significantly elevated in all obese subgroups in their work in contrast to our results [60]. In line with the findings of Schinzari et al., our results suggest a different mechanism related to the dysregulation of ANGPTL3 and ANGPTL4 in patients with excessive fat mass. It is worth mentioning here that previous works measuring ANGPTL3 levels in insulin resistance states such as obesity and T2D have produced conflicting results. Most studies report increased circulation of ANGPTL3 in obese or T2D patients [60,61], while other studies have found that ANGPTL3 levels are not higher in obese subjects than in controls [62].

### Study Limitation

Our study also had the following limitations: the case–control design of the study; the moderate size of same-sex population; only hsCRP was measured as a significant marker of inflammation; using BMI as a marker of obesity, as it does not make it possible to distinguish between fat tissue and lean tissue; there are different criteria for defining MHO individuals, and the occurrence is dependent on the definition used.

## 5. Conclusions

Our results show that MHO subjects are more similar to MUO subjects than those with MHNW considering metabolic disorders. Despite the absence of classic metabolic disorders, our results, which are based on obesity and diabesity markers, indicate this subclinical difference between MHO and MUO. When it comes to distinguishing between the MHO phenotype and the MUO phenotype, our analyses show that only adipsin is associated with the MHO phenotype and not associated with the MUO phenotype. In addition, markers such as ghrelin and PAI-1 are only associated with MUO phenotype than MHO phenotype. It is necessary to conduct prospective studies to assess the conversion of MHO to MUO and the importance of the biomarkers we selected in this process. Our proposed biomarkers should be verified in the future on a larger study group in terms of sensitivity and specificity. Earlier identification of obesity-related or metabolic disease risk factors may contribute to the faster implementation of effective treatment. The treatment of obesity and comorbidities is a significant medical problem that does not always bring the expected results in all patients treated. Therefore, it is essential to select the obese patients who will benefit most from early intervention, such as lifestyle changes or pharmacological treatment.

## Figures and Tables

**Table 1:** Epidemiologic characteristics of study participants according to metabolic status.

Variables	MHNW (*n* = 49)	MHO (*n* = 27)	MUO (*n* = 22)	*p*-Value *	*p*-Value **
Age (years)	30.96 [27.30–32.42]	29.88 [26.61–32.67]	31.82 [28.12–33.10]	>0.05	>0.05
Currently smoking (vs. past smoker or non-smoker) (%)	10 (20.41)	4 (14.81)	6 (27.27)	>0.05	>0.05
Alcohol consumption (%)	42 (85.71)	21 (77.77)	18 (81.81)	>0.05	>0.05
Family history of P-CAD (%)	21 (42.86)	18 (66.67)	16 (72.72)	<0.05	>0.05
Family history of DM (%)	6 (12.24)	4 (14.81)	5 (22.73)	>0.05	>0.05
Less than six hours of sleep per night during weekdays (%)	23 (46.94)	12 (44.44)	8 (36.36)	>0.05	>0.05
Less than six hours of sleep per night during the weekends (%)	4 (8.16)	3 (11.11)	1 (4.54)	>0.05	>0.05
Low physical activity level (%)	18 (36.74)	10 (37.04)	15 (68.18)	>0.05	>0.05

Continuous variables are expressed as the median (25th–75th percentiles) or as absolute (*n*) and relative (%) frequencies. MHNW: metabolically healthy normal weight; MHO: metabolically healthy obese; MUO: metabolically unhealthy obese; DM: diabetes mellitus; P-CAD: premature coronary artery disease; * *p*-value of the Kruskal–Wallis rank sum test (continuous variable) or chi-squared test (categorical variable); ** *p*-value of the Jonckheere–Terpstra test (continuous variable) or Cochran–Armitage test (categorical variable).

**Table 2:** Anthropometric and biochemical characteristics of study participants according to metabolic status.

Variables	MHNW (*n* = 49)	MHO (*n* = 27)	MUO (*n* = 22)	*p*-Value *	*p*-Value **
BMI [kg/m^2^]	23.36 [21.78–24.10]	31.38 [30.63–33.05]	34.02 [33.03–37.02]	<0.001	<0.001
WHR [cm]	0.84 [0.81–0.88]	0.93 [0.88–0.96]	1.00 [0.97–1.02]	<0.001	<0.001
VAI	0.69 [0.49–0.82]	1.30 [1.09–1.77]	3.77 [2.53–5.31]	<0.001	<0.001
SBP [mmHg]	128.00 [120.00–134.50]	135.00 [128.00–144.00]	136.00 [132.00–157.00]	<0.001	<0.001
DBP [mmHg]	80.00 [73.75–84.00]	85.00 [77.50–89.00]	90.00 [82.00–95.00]	<0.01	<0.001
TC [mmol/L]	4.80 [4.27–5.30]	5.41 [4.74–6.17]	5.77 [5.04–6.87]	<0.001	<0.001
HDL-C [mmol/L]	1.58 [1.40–1.74]	1.19 [1.15–1.44]	1.01 [0.89–1.15]	<0.001	<0.001
HDL%	33.00 [29.00–38.00]	24.00 [20.00–27.50]	17.50 [13.25–21.75]	<0.001	<0.001
LDL-C [mmol/L]	2.84 [2.49–3.43]	3.71 [2.94–4.25]	3.76 [3.13–4.27]	<0.001	<0.001
TG [mmol/L]	0.80 [0.64–1.03]	1.30 [1.13–1.56]	2.76 [1.94–3.76]	<0.001	<0.001
Lp(a) [nmol/L]	17.00 [5.00–48.00]	9.00 [4.50–23.50]	9.50 [3.25–102.75]	>0.05	>0.05
apoA1 [g/L]	1.57 [1.49–1.71]	1.44 [1.41–1.69]	1.46 [1.32–1.61]	<0.05	<0.01
apoB [g/L]	0.85 [0.76–1.05]	1.12 [0.93–1.25]	1.23 [1.05–1.42]	<0.001	<0.001
HbA1c (%)	4.90 [4.80–5.20]	5.10 [4.85–5.35]	5.10 [5.00–5.20]	<0.05	<0.01
Glucose [mmol/L]	5.00 [4.70–5.30]	5.20 [4.85–5.60]	5.35 [5.10–5.90]	<0.01	<0.001
HOMA-IR	1.46 [1.20–1.70]	2.76 [2.17–3.34]	3.51 [1.63–4.49]	<0.001	<0.001
hsCRP [mg/dL]	0.66 [0.46–1.04]	1.21 [0.62–1.67]	1.73 [1.20–2.25]	<0.001	<0.001
Bilirubin [µmol/L]	13.00 [9.90–17.60]	11.00 [7.90–16.35]	9.05 [6.93–14.03]	<0.05	<0.01
Uric acid [µmol/L]	314.00 [296.00–367.00]	387.00 [353.00–409.50]	403.50 [364.75–439.00]	<0.001	<0.001
Fibrinogen [mg/dL]	248.00 [220.00–297.00]	256.00 [234.00–278.50]	303.00 [260.25–334.00]	<0.01	<0.01
Ceruloplasmin [g/L]	0.20 [0.18–0.22]	0.22 [0.21–0.23]	0.23 [0.21–0.25]	<0.001	<0.001

Continuous variables are expressed as the median (25th–75th percentiles). MHNW: metabolically healthy normal weight; MHO: metabolically healthy obese; MUO: metabolically unhealthy obese; BMI: body mass index; WHR: waist–hip ratio; VAI: visceral adiposity index; SBP: systolic blood pressure; DBP: diastolic blood pressure; TC: total cholesterol; HDL-C: high-density lipoprotein cholesterol; LDL-C: low-density lipoprotein cholesterol; TG: triglycerides, Lp(a): lipoprotein(a); apoA1: apolipoprotein A1; apoB: apolipoprotein B; HbA1c: glycated haemoglobin; HOMA-IR: homeostasis model assessment of insulin resistance; hsCRP: high-sensitivity C-reactive protein; * *p*-value of the Kruskal–Wallis rank sum test; ** *p*-value of the Jonckheere–Terpstra test.

**Table 3:** Levels of selected markers related to diabesity and obesity among subjects according to metabolic status.

Variables	MHNW (*n* = 49)	MHO (*n* = 27)	MUO (*n* = 22)	*p*-Value *	*p*-Value **
Adiponectin [mg/L]	15.45 [8.85–26.03]	7.18 [5.80–12.00]	4.81 [3.10–8.89]	<0.001	<0.001
Adipsin [mg/L]	1.37 [0.92–1.96]	0.98 [0.81–1.24]	1.03 [0.85–1.51]	<0.05	<0.05
Leptin [pg/mL]	783.15 [409.82–1357.25]	6086.88 [2505.89–9811.66]	8464.22 [7045.08–13,405.24]	<0.001	<0.001
Leptin/Adiponectin ratio	43.92 [27.47–108.77]	577.85 [315.54–1374.92]	2039.48 [843.05–3053.01]	<0.001	<0.001
Visfatin [pg/mL]	14,961.90 [12,397.98–20,576.90]	14,809.15 [13,715.20–19,280.44]	15,319.60 [12,696.9–17,003.08]	>0.05	>0.05
Resistin [pg/mL]	5933.41 [3821.07–8860.00]	5400.22 [4198.69–8922.45]	6329.89 [4354.88–9574.26]	>0.05	>0.05
Ghrelin [pg/mL]	541.19 [401.98–847.85]	476.62 [333.62–623.31]	401.83 [304.74–481.09]	<0.01	<0.001
GIP [pg/mL]	157.28 [121.10–168.27]	151.70 [86.57–212.35]	162.82 [129.16–198.10]	>0.05	>0.05
C-peptide [pg/mL]	525.64 [416.93–742.86]	1103.01 [872.32–2310.21]	2453.77 [1471.87–3504.04]	<0.001	<0.001
GLP-1 [pg/mL]	245.56 [155.74–324.58]	205.62 [124.78–285.86]	235.37 [185.01–307.65]	>0.05	>0.05
Glucagon [pg/mL]	1445.99 [1159.42–1828.44]	1540.74 [1224.63–1744.17]	1480.60 [1226.07–1768.03]	>0.05	>0.05
Insulin [pg/mL]	237.50 [183.22–274.48]	449.05 [321.08–517.38]	494.55 [251.64–674.10]	<0.001	<0.001
PAI-1 [pg/mL]	77,353.49 [50,801.99–111,687.54]	86,828.42 [48,879.62–133,191.15]	92,513.11 [82,278.29–147,186.72]	>0.05	>0.05
ANGPTL3 [ng/mL]	56.03 [46.44–71.64]	68.30 [53.59–88.06]	75.14 [58.74–95.57]	<0.05	<0.01
ANGPTL4 [ng/mL]	181.17 [151.17–225.22]	188.68 [121.99–243.08]	185.71 [125.13–259.42]	>0.05	>0.05

Continuous variables are expressed as the median (25th–75th percentiles). MHNW: metabolically healthy normal weight; MHO: metabolically healthy obese; MUO: metabolically unhealthy obese; GIP: gastric inhibitory peptide; GLP-1: glucagon-like peptide-1; PAI-1: plasminogen activator inhibitor-1.; ANGPTL3: angiopoietin-like 3 protein; ANGPTL4: angiopoietin-like 4 protein; * *p*-value of the Kruskal–Wallis rank sum test; ** *p*-value of the Jonckheere–Terpstra test.

**Table 4:** Univariate linear regression analyses of the effects of markers related to obesity and diabesity on the metabolic health status.

Dependent Variable	Independent Variable	OR	95% CI	*p*-Value
Adiponectin	MHO	0.56	0.38–0.82	<0.01
	MUO	0.37	0.25–0.56	<0.001
Adipsin	MHO	0.76	0.61–0.94	<0.05
	MUO	0.85	0.68–1.07	>0.05
Leptin	MHO	6.75	4.69–9.73	<0.001
	MUO	12.52	8.47–18.51	<0.001
Leptin/Adiponectin ratio	MHO	0.08	0.05–0.14	<0.001
	MUO	0.03	0.02–0.05	<0.001
Visfatin	MHO	1.05	0.91–1.21	>0.05
	MUO	0.96	0.82–1.12	>0.05
Resistin	MHO	0.96	0.72–1.27	>0.05
	MUO	1.02	0.75–1.38	>0.05
Ghrelin	MHO	0.82	0.65–1.03	>0.05
	MUO	0.63	0.49–0.80	<0.001
GIP	MHO	0.94	0.77–1.15	>0.05
	MUO	1.01	0.81–1.26	>0.05
C-peptide	MHO	2.40	1.79–3.22	<0.001
	MUO	4.31	3.15–5.90	<0.001
GLP-1	MHO	0.82	0.64–1.05	>0.05
	MUO	1.02	0.78–1.32	>0.05
Glucagon	MHO	1.07	0.92–1.23	>0.05
	MUO	1.01	0.86–1.17	>0.05
Insulin	MHO	1.67	1.35–2.07	<0.001
	MUO	1.88	1.49–2.36	<0.001
PAI-1	MHO	1.10	0.75–1.62	>0.05
	MUO	1.72	1.14–2.60	<0.05
ANGPTL3	MHO	1.23	1.01–1.49	<0.05
	MUO	1.28	1.05–1.58	<0.05
ANGPTL4	MHO	1.02	0.85–1.22	>0.05
	MUO	0.99	0.82–1.19	>0.05

OR: odds ratio; CI: confidence interval; MHO: metabolically healthy obese; MUO: metabolically unhealthy obese; GIP: gastric inhibitory peptide; GLP-1: glucagon-like peptide-1; PAI-1: plasminogen activator inhibitor-1; ANGPTL3: angiopoietin-like 3 protein; ANGPTL4: angiopoietin-like 4 protein.

**Table 5:** Multivariate linear regression analyses of the effects of markers related to obesity and diabesity on the metabolic health status.

Dependent Variable	Covariates	Independent Variables	OR	95% CI	*p*-Value
Adiponectin	Age, smoking, alcohol consumption	MHO	0.56	0.38–0.83	<0.01
		MUO	0.38	0.25–0.57	<0.001
Adipsin	Age, smoking, alcohol consumption	MHO	0.75	0.61–0.93	<0.05
		MUO	0.88	0.70–1.11	>0.05
Leptin	Age, smoking, alcohol consuption	MHO	6.69	4.62–9.70	<0.001
		MUO	12.52	8.40–18.65	<0.001
Leptin/Adiponectin ratio	Age, smoking, alcohol consumption	MHO	0.08	0.05–0.14	<0.001
		MUO	0.03	0.02–0.05	<0.001
Ghrelin	Age, smoking, alcohol consumption	MHO	0.81	0.65–1.02	>0.05
		MUO	0.65	0.51–0.83	<0.001
C-peptide	Age, smoking, alcohol consumption	MHO	2.40	1.79–3.24	<0.001
		MUO	4.21	3.06–5.80	<0.001
Insulin	Age, smoking, alcohol consumption	MHO	1.67	1.34–2.07	<0.001
		MUO	1.88	1.49–2.38	<0.001
PAI-1	Age, smoking, alcohol consumption	MHO	1.07	0.73–1.58	>0.05
		MUO	1.76	1.16–2.67	<0.01
ANGPTL3	Age, smoking, alcohol consumption	MHO	1.23	1.01–1.50	<0.05
		MUO	1.27	1.03–1.57	<0.05

OR: odds ratio; CI: confidence interval; MHO: metabolically healthy obese; MUO: metabolically unhealthy obese; PAI-1: plasminogen activator inhibitor-1; ANGPTL3: angiopoietin-like 3 protein.

## Data Availability

The data presented in this study are available on request from the corresponding author.
